# KISSPLICE: de-novo calling alternative splicing events from RNA-seq data

**DOI:** 10.1186/1471-2105-13-S6-S5

**Published:** 2012-04-19

**Authors:** Gustavo AT Sacomoto, Janice Kielbassa, Rayan Chikhi, Raluca Uricaru, Pavlos Antoniou, Marie-France Sagot, Pierre Peterlongo, Vincent Lacroix

**Affiliations:** 1 INRIA Grenoble Rhône-Alpes, France; 2 Université de Lyon, F-69000, Lyon; Université Lyon 1; CNRS, UMR5558, Laboratoire de Biométrie et Biologie Evolutive, F-69622, Villeurbanne, France; 3 Centre de recherche INRIA Rennes - Bretagne Atlantique, IRISA, Campus universitaire de Beaulieu, Rennes, France; 4 INRA UMR118, Amélioration des Plantes et Biotech. Végétales, Rennes, France

## Abstract

**Background:**

In this paper, we address the problem of identifying and quantifying polymorphisms in RNA-seq data when no reference genome is available, without assembling the full transcripts. Based on the fundamental idea that each polymorphism corresponds to a recognisable pattern in a De Bruijn graph constructed from the RNA-seq reads, we propose a general model for all polymorphisms in such graphs. We then introduce an exact algorithm, called KISSPLICE, to extract alternative splicing events.

**Results:**

We show that KISSPLICE enables to identify more correct events than general purpose transcriptome assemblers. Additionally, on a 71 M reads dataset from human brain and liver tissues, KISSPLICE identified 3497 alternative splicing events, out of which 56% are not present in the annotations, which confirms recent estimates showing that the complexity of alternative splicing has been largely underestimated so far.

**Conclusions:**

We propose new models and algorithms for the detection of polymorphism in RNA-seq data. This opens the way to a new kind of studies on large HTS RNA-seq datasets, where the focus is not the global reconstruction of full-length transcripts, but local assembly of polymorphic regions. KISSPLICE is available for download at http://alcovna.genouest.org/kissplice/.

## Background

Thanks to recent technological advances, sequencing is no longer restricted to genomes and can now be applied to many new areas, including the study of gene expression and splicing. The so-called RNA-seq protocol consists in applying fragmentation and reverse transcription to a RNA sample followed by sequencing the ends of the resulting cDNA fragments. The short sequencing reads then need to be reassembled in order to get back to the initial RNA molecules. A lot of effort has been put on this assembly task [2], whether in the presence or in the absence of a reference genome but the general goal of identifying and quantifying all RNA molecules initially present in the sample remains hard to reach. The main challenge is certainly that reads are short, and can therefore be ambiguously assigned to multiple transcripts. In particular, in the case of alternative splicing (AS for short), reads stemming from constitutive exons can be assigned to any alternative transcript containing this exon. Finding the correct transcript is often not possible given the data we have, and any choice will be arguable. As pointed out in Martin and Wang's review [2], reference-based and de novo assemblers each have their own limitations. Reference-based assemblers depend on the quality of the reference while only a small number of species currently have a high-quality reference genome available. De novo assemblers implement reconstruction heuristics which may lead them to miss infrequent alternative transcripts while highly similar transcripts are likely to be assembled into a single transcript. We argue here that it is not always necessary to aim at the difficult goal of assembling full-length molecules. Instead, identifying the variable parts between molecules (polymorphic regions) is already very valuable and does not require to solve the problem of assigning a constitutive read to the correct transcript. We therefore focus in this paper on the simpler task of identifying polymorphisms in RNA-seq data. Three kinds of polymorphisms have to be considered: i) AS (alternative splicing) that produces several alternative transcripts for a same gene, ii) SNPs (single nucleotide polymorphism) that may also produce several transcripts for a same gene whenever they affect transcribed regions, and iii) approximate tandem repeats which affect the number of copies of tandem repeats. Our contribution in this paper is double: we first give a general model which captures these three types of polymorphism by linking them to characteristic structural patterns called "bubbles" in the De Bruijn graph (DBG for short) built from a set of RNA-seq reads, and second, we propose a method dedicated to the problem of identifying AS events in a DBG, including read-coverage quantification. We notice here that only splicing events but not transcriptional events, such as alternative start and polyadenylation sites, are covered by our method.

The identification of bubbles or bulges in DBG has been studied before in the context of genome assembly [3-5]. However, the purpose in these works was not to enumerate these patterns, but "only" to remove them from the graph. Additionally, since in these applications, the patterns correspond to SNPs and sequencing errors, the authors only considered paths of length smaller than a constant.

More recently, ad-hoc enumeration methods have been proposed but are restricted to non-branching bubbles [6], *i*.*e*., each vertex from the bubble has in-degree and out-degree 1, except for the extremities of the bubble.

Extracting AS events from a splicing graph has been studied before [7] but a significant difference between splicing graphs and De Bruijn graphs is that in the former, nodes are genomically ordered (through the use of a reference annotated genome) therefore leading to a DAG, whereas DBGs are general graphs, that furthermore do not require any additional information to be built.

When no reference genome is available, efforts have focused on assembling the full-length RNA molecules, not the variable parts which are our interest here. Most RNA-seq assemblers [8-10] do rely on the use of a DBG, but, since the primary goal of an assembler is to produce the longest contigs, heuristics are applied, such as tip or bubble removal, in order to linearise the graph. The application of such heuristics results in a loss of information which may in fact be crucial if the goal is to study polymorphism.

To our knowledge, this work is the first attempt to characterise polymorphism in RNA-seq data without assembling full-length transcripts. We stress that it is not a general purpose transcriptome assembler and when we benchmark it against such methods, we only focus on the specific task of AS event calling. Finally, our method can be used with a single or multiple RNA-seq experiments and our quantification module outputs a coverage (reads per nt) for both the shorter and the longer isoform(s) of each AS event, in each experiment.

The paper is organised as follows. We first present the model (Section "*De-Bruijn graph models*") linking structures of the DBG for a set of RNA-seq reads to polymorphism, and then introduce a method, that we call KISSPLICE, for identifying DBG structures associated with AS events (Section "*The *KISSPLICE*algorithm*"). We show in Section "*Results*" the results of using KISSPLICE compared with other methods on simulated and real data.

## Methods

### De-Bruijn graph models

#### De-Bruijn graph

**Figure 1:**
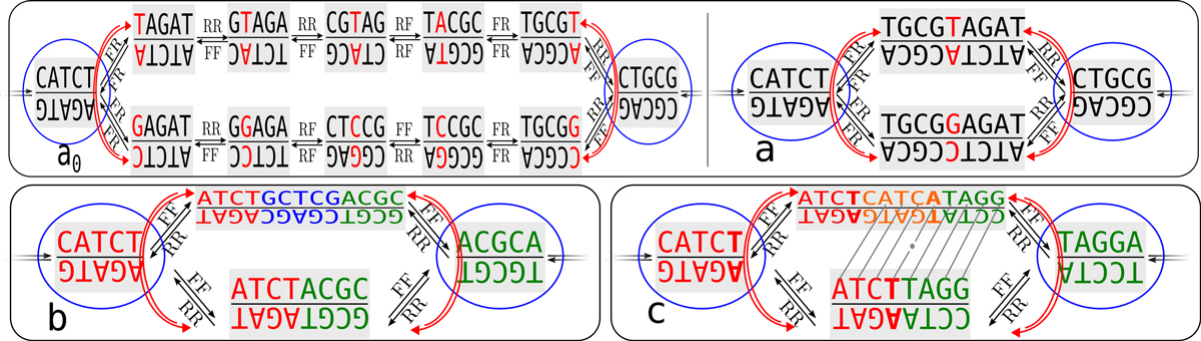
**De Bruin graphs**. Part of non-compressed **(a_0_) **and compressed **(a, b, c) **de Bruijn graphs (*k *= 5). Each node contains a word (upper text of each node) and its reverse complement (lower text of each node). In the uncompressed graph, the word is a *k*-mer. Encircled nodes are switching with respect to red paths (pointed out by red arrows). **(a_0_, a) **Bubble due to a substitution (red letter). Starting from the forward strand in the leftmost (switching) node would generate the sequences CATCT A CGCAG (upper path) and CATCT C CGCAG (lower path). **(b) **Bubble due to the skipped exon GCTCG (blue sequence). This bubble is generated by the sequences CATCT ACGCA and CATCT GCTCG ACGCA. **(c) **Bubble due to an inexact tandem repeat. This bubble is generated by the sequences CATCT TAGGA and CATCT CATCA TAGGA, where CATC**T **CATC**A **is an inexact tandem repeat.

DBGs were first used in the context of genome assembly in 2001 by Pevzner *et al*. [11]. In 2007, Medvedev *et al*. [12] modified the definition to better model DNA as a double stranded molecule. In such a context, a DBG is a bidirected multigraph, each node *N *storing a sequence *w *and its reverse complement $\bar{w}$. The sequence *w*, denoted by *F*(*N*), is the forward sequence of *N*, while $\bar{w}$, denoted by *R*(*N*), is the reverse complement sequence of *N*. An arc exists from node *N*_1 _to node *N*_2 _if the suffix of length *k *- 1 of *F*(*N*_1_) or *R*(*N*_1_) overlaps perfectly with the prefix of *F*(*N*_2_) or *R*(*N*_2_). Each arc is labelled with a string in {*FF, RR, FR, RF*}. The first letter of the arc label indicates which of *F*(*N*_1_) or *R*(*N*_1_) overlaps *F*(*N*_2_) or *R*(*N*_2_), this latter choice being indicated by the second letter. Because of reverse complements, there is an even number of arcs in the DBG: if there is an arc from *N*_1 _to *N*_2 _then, necessarily, there is an arc from *N*_2 _to *N*_1 _(*e*.*g*. if the first arc has label *FF *then the second has label *RR*). Examples of DBGs are presented in Figure 1.

##### Definition 1 (Valid path)

*The traversal of a node is said to be valid if the rightmost label (F or R) of the arc entering the node is equal to the leftmost label of the arc leaving the node*.

*A path in the graph is valid if for each node involved in the path, its traversal is valid, that is, each pair of adjacent arcs in the path are labelled, respectively, XY and Y Z with X, Y, Z *∈ {*R, F*}.

For instance, for any graph shown in Figure 1, the path starting from the leftmost encircled node, going by the upper path to the rightmost encircled node is valid. A DBG can be compressed without loss of information by merging simple nodes. A *simple *node denotes a node linked to at most two other nodes. Two adjacent simple nodes are merged into one by removing the redundant information. A valid path composed by *i *> 1 simple nodes is compressed into one node storing a sequence of length *k *+ (*i - *1) as each node adds one new character to the first node. Figure 1.a represents the compressed DBG shown in Figure 1.a0. In the remaining of the paper, we denote by cDBG a compressed DBG.

#### Bubble patterns in the cDBG

Polymorphisms (*i*.*e*. variable parts) in a transcriptome or a genome, correspond to recognisable patterns in the cDBG, which we call a **bubble**. Intuitively, the variable parts will correspond to alternative paths and the common parts will correspond to the beginning and end points of these paths. We now formally define the notion of bubble, taking carefully into account the bi-directed and arc labeled nature of the cDBG.

##### Definition 2 (Node switching with respect to a path)

*A node is switching with respect to a path if this path is invalid during the traversal of this node*.

##### Definition 3 (Bubble)

*In the cDBG, a bubble is a simple cycle involving at least three distinct nodes such that exactly two nodes SN_left _and SN_right _are switching w.r.t. the path of the cycle. By definition, two valid paths exist between these two switching nodes. In the remaining of the paper, we refer to these two paths as the paths of the bubble. If they differ in length, we refer to, respectively, the longer and the shorter path of the bubble*.

Figure 1 presents four bubbles. For each one, their switching nodes are encircled in blue.

In general, any process generating patterns *asb *and *as'b *in the sequences, with *a, b, s, s' *∈ Σ*, |*a*| ≥ *k*, |*b*| ≥ *k *and *s *and *s' *not sharing any *k*-mer, creates a bubble in the cDBG. Indeed, all *k*-mers entirely contained in *a *(resp. *b*) compose the node *SN_left _*(resp. *SN_right_*). Since |*a*| ≥ *k *and *s *≠ *s'*, there is at least one pair of k-mers, one in as and the other in *as'*, sharing the *k *- 1 prefix and differing by the last letter, thus creating a branch in *SN_left _*from which the two paths in the bubble diverge. The same applies for *sb, s'b *and *SN_right_*, where the paths merge again. All *k*-mers contained in *s *(resp. *s'*) and in the junctions *as *and *sb *(resp. *as' *and *s'b*) compose the paths of the bubble. In the case of AS events and approximate tandem repeats, *s *is empty and the shorter path is composed of *k*-mers covering the junction *ab*.

This model is general as it captures SNPs, approximate tandem repeats and AS events, as shown in Figure 1. The main focus of the algorithm we present in this paper is the detection of bubbles generated by AS events.

#### Bubbles generated by AS events

**Figure 2:**
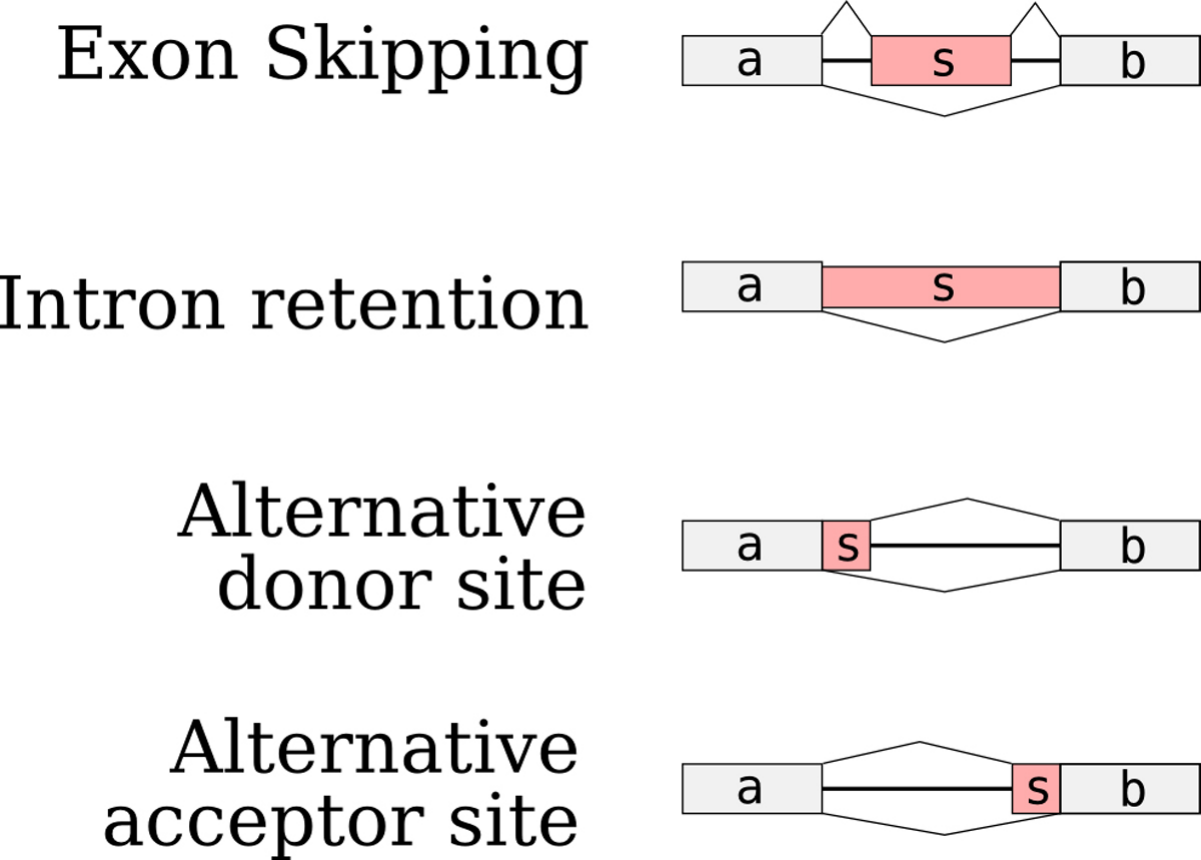
**AS events generating a bubble in the DBG**. All these events create a bubble in the DBG or cDBG, in which the shorter path is composed by *k*-mers covering the *ab *junction. This path, composed by *k *- 1 nodes in the DBG, is compressed into a sequence of length 2*k *- 2 in the cDBG (Figure 1.b).

A single gene may give rise to multiple alternative spliceforms through the process of AS. Alternative spliceforms differ locally from each other by the inclusion or exclusion of subsequences. These subsequences may correspond to exons (exon skipping), exon fragments (alternative donor or acceptor sites) or introns (intron retention) as shown in Figure 2. Observe that alternative start and polyadenylation sites, which are not considered as AS events but as transcriptional events, are not taken into account in this work. A splicing event corresponds to a local variation between two alternative transcripts. It is characterised by two common sites (called *a *and *b *in the examples given in Figure 2) and a variable part (called *s *Figure 2). In the cDBG, the common sites correspond to the switching nodes and the variable part to the longer path. As there are *k *- 1 *k*-mers at the junction between the two common sites *a*.*b*, the shorter path is composed of at most *k *- 1 *k*-mers, *i*.*e*. represents a path of length at most 2*k *- 2 in the cDBG. An example is given in Figure 1.b.

The shorter path of a bubble generated by an AS event has length exactly 2*k *- 2 iff (i) the last nucleotide (nt for short) of the variable part is distinct from the last nt of the left switching node, and (ii) the first nt of the variable part is distinct from the first nt of the right switching node. Otherwise, the two alternative paths join (case (i)) or diverge (case (ii)) earlier and the shorter path may be smaller. In human, 99% of the annotated exon skipping events yield a bubble with a shorter path length between 2*k *- 8 and 2*k *- 2.

#### Bubbles generated by SNPs and approximate tandem repeats

Polymorphism at the genomic level will necessarily also be present at the transcriptomic level whenever it affects transcribed regions. Two major kinds of polymorphism can be observed at the genomic level: SNPs and approximate tandem repeats. As shown in Figure 1, these two types of polymorphism also generate bubbles in the cDBG.

However, these bubbles have characteristics which enable to differentiate them from bubbles generated by AS events. Indeed, bubbles generated by SNPs exhibit two paths of length exactly 2*k *- 1, which is larger than 2*k *- 2, the maximum size of the shorter path in a bubble generated by an AS event.

Approximate tandem repeats may generate bubbles with a similar path length as bubbles generated by splicing events, but the sequences of the paths exhibit a clear pattern which can be easily identified: the longer path contains an inexact repeat. More precisely, as outlined in Figure 1.c, it is sufficient to compare the shorter path with one of the ends of the longer path.

Finally, genomic insertions or deletions (indels for short) may also generate bubbles with similar path lengths as bubbles generated by splicing events. In this case, the difference of length between the two paths is usually smaller (less than 3 nt for 85% of indels in human transcribed regions [13] whereas it is more than 3 nt for 99% of AS events). In our method, when the difference of path lengths is strictly below 3, we classify the bubble as an indel. Otherwise, we do not decide, which means that a fraction of the bubbles we report as AS events will correspond to indels. Note that this classification is a simple suggestion. We encourage users to affine results by considering species specificity and by applying coverage criterion.

In the following, we focus on bubbles generated by AS events. We do provide as a collateral result three additional collections of bubbles: one corresponding to putative SNPs, one to short indels, and one to putative approximate tandem repeats. The post-treatment of these collections to discard false positives caused by sequencing errors is beyond the scope of this paper.

### The KISSPLICE algorithm

The KISSPLICE algorithm detects in the cDBG all the bubble patterns generated by AS events, *i*.*e*. the bubbles having a shorter path of length at most 2*k *- 2. Essentially, the algorithm enumerates all the cycles verifying the two following criteria: i) the path obtained by following all the nodes of the cycle contains exactly two nodes that are switching for this path, and ii) the length of the shorter path linking the two switching nodes must be no longer than 2*k *- 2. Further criteria are applied to make the algorithm more efficient without loss of information, and to eliminate polymorphism events that do not correspond to AS. Since the number of cycles in a graph may be exponential with the size of the graph, the naive approach of enumerating all cycles of the cDBG and verifying which of them satisfy our conditions is only viable for very small cases.

Nonetheless, KISSPLICE is able to enumerate a potentially exponential number of bubbles for real-sized data in very reasonable time and memory. This is in part due to the fact that, previous to cycle enumeration, the graph is pre-processed in a way that, along with the pruning criteria of Step 4 (see below), is responsible for a good performance in practice.

KISSPLICE is indeed composed of six main steps which are described next. The pre-processing just mentioned corresponds to Step 2. As far as we know, it is the first time it is used in conjunction with cycle enumeration.

**Step 1**. Construction of the cDBG of the reads of one or several RNA-seq experiments. Each node contains the coverage of the corresponding *k*-mer in each experiment. In order to get rid of most of the sequencing errors, nodes with a minimal coverage of 1 may be removed.

**Step 2**. Biconnected component (BCC for short) decomposition. A connected undirected graph is *biconnected *if it remains connected after the removal of any vertex. A BCC of an undirected graph is a maximal biconnected subgraph. Moreover, it is possible to show that the BCCs of an undirected graph form a partition of the edges with two important properties: every cycle is contained in exactly one BCC, and every edge not contained in a cycle forms a singleton BCC. Applying on the underlying undirected graph of the cDBG Tarjan's lowpoint method [14] which performs a modified depth-first search traversal of the graph, Step 2 detects all BCCs, and discards all singleton ones that could not contain any bubble. Without modifying the results, this considerably reduces the memory footprint and the computation time of the whole process. To give an idea of the effectiveness of this step, the cDBG of a 5 M dataset had 1.7 M nodes, but the largest BCC only 2961 nodes.

**Step 3**. Four-nodes compression. Single substitution events (SNPs, sequencing errors) generate a large number of cycles themselves included into bigger ones, creating a combinatorial explosion of the number of possible bubbles. This step of KISSPLICE detects and compresses all bubbles composed by just four nodes: two switching nodes and two *non-branching internal nodes *each storing equal length sequences differing by just one position. Figure 1.a shows an example of a four-nodes bubble. Four-nodes bubbles are output as potential SNPs and then reduced to a three-nodes path. The two non-branching internal nodes are merged into one, storing a consensus sequence where the unique substitution is replaced by N.

**Step 4**. Bubbles enumeration. The cycles are detected in the cDBG using a backtracking procedure [15] augmented with two pruning criteria. The exploration of one cycle is stopped if the path contains more than two nodes that are switching relative to the path that is being followed, or the length of the shorter path is bigger than 2*k *- 2. This approach has the same theoretical time complexity of Tiernan's algorithm for cycle enumeration [15], which is worse than Tarjan's [16] polynomial delay algorithm but it appears to be not immediate how to use the pruning criteria with the latter while preserving its theoretical complexity. We however were able to show that in practice, the pruning criteria are very effective for the type of instances we are dealing with. Indeed, we compared the three following implementations on a 1 M reads dataset: i) Tiernan ii) Tarjan iii) Tiernan with prunings (our method). The results clearly showed that, while Tarjan (22 min) outperforms Tiernan (32 min), both are clearly outperformed when the prunings are used (4 s).

**Step 5**. Results filtration and classification. The two paths of each bubble are aligned. If the whole of the shorter path aligns with high similarity to the longer path, we decide that the bubble is due to an approximate tandem repeat (see Section "*Bubbles generated by SNPs and approximate tandem repeats*"). After this alignment, a bubble is classified either as an AS event, an approximate tandem repeat, or a small indel (less than 3 nt).

**Step 6**. Read coherence and coverage computation. Reads from each input dataset are mapped to each path of the bubble. If at least one nucleotide of a path is covered by no read, the bubble is said to be not read-coherent and is discarded. The coverage of each position of the bubble corresponds to the number of reads overlapping this position. Border effects are handled in the following way: reads mapping to the extremity of a path with less than *k *bases are discarded. This results in a systematic under-estimation of the coverage of the extremities of the path. Under a simple assumption of locally uniform coverage, this can be counter-balanced by multiplying the coverage of each of the *k *- 1 external positions by a correction factor of $\frac{L}{L-i}$, with *L *the read size and *i *the distance to the first non biased position. This correction is possible because the paths considered correspond to internal transcript sequences, not to a transcription start or end.

## Results

### Simulated data

In order to assess the sensitivity and specificity of our approach, we simulated the sequencing of genes for which we are able to control the number of alternative transcripts. We show that the method is indeed able to recover AS events whenever the alternative transcripts are sufficiently expressed. For our sensitivity tests, we used simulated RNA-seq single end reads (75 bp) with sequencing errors. We first tested a pair of transcripts with a 200 nt skipped exon. Simulated reads were obtained with MetaSim [17] which is a reference software for simulating sequencing experiments. As in real experiments, it produces heterogeneous coverage and authorises to use realistic error models.

In order to find the minimum coverage for which we are able to work, we created datasets for several coverages (from 4× to 20×, which corresponds to 60 to 300 Reads Per Kilobase or RPK for short), with 3 repetitions for each coverage, and tested them with different values of *k *(*k *= 13, ...41). The purpose of using 3 repetitions for each coverage was to obtain results which did not depend on irreproducible coverage biases. For coverages below 8× (120 RPK), KISSPLICE found the correct event in some but not all of the 3 tested samples. The failure to detect the event was due to the heterogenous and thus locally very low coverage around the skipped exon, *e*.*g*. some nt were not covered by any read or the overlap between the reads was smaller than *k*-1. Above 8× (120 RPK), KISSPLICE detected the correct exon skipping event in all samples.

For each successful test, there was a maximal value *k_max _*for k above which the event was not found, and a minimal value *k_min _*below which KISSPLICE also reported false positive events. Indeed, if *k *is too small, then the pattern *ab, as 'b*, with |*a*| ≥ *k*, |*b*| ≥ *k *is more likely to occur by chance in the transcripts, therefore generating a bubble in the DBG. Between these two thresholds, KISSPLICE found only one event: the correct one. The values of *k_min _*and *k_max _*are clearly dependent on the coverage of the gene. At 8× (120 RPK), the 200 nucleotides exon was found between *k_min _*= 17 and *k_max _*= 29. At 20× (300 RPK), it was found for *k_min _*= 17 and *k_max _*= 39. We performed similar tests on other datasets, varying the length of the skipped exon. As expected, if the skipped exon is shorter (longer), KISSPLICE needed a lower (higher) coverage to recover it.

Since KISSPLICE is, to our knowledge, the first method able to call AS events without a reference genome, it cannot be easily benchmarked against other programs. Here, we compare it to a general purpose transcriptome assembler, Trinity [8]. Both methods are compared only on the specific task of AS event calling. The current version of Trinity being restricted to a fixed value of *k *= 25, we systematically verified that this value was included in [*k_min_, k_max_*].

We found out that Trinity was able to recover the AS event in all 3 samples only when the coverage was above 18× (270 RPK), which clearly shows that KISSPLICE is more sensitive for this task. This can be explained by the fact that TRINITY uses heuristics which consist in discarding a *k*-mer in the DBG whenever it is 20 times less frequent than an alternative *k*-mer branching at the same location in the DBG.

All these results were obtained using a minimal k-mer coverage (mkC for short) of 1. We also tested with mkC = 2 (*i*.*e*. *k*-mers present only once in the dataset are discarded), leading to the same main behaviour. We noticed however a loss in sensitivity for both methods, but a significant gain in the running time. KISSPLICE found the event in all 3 samples for a coverage of 12× (180 RPK) which remains better than the sensitivity of Trinity for mkC = 1.

### Real data

We further tested our method on RNA-seq data from human. Even though we do not use any reference genome in our method, we applied it to cases where an annotated reference genome is indeed available in order to be able to assess if our predictions are correct.

**Figure 3:**
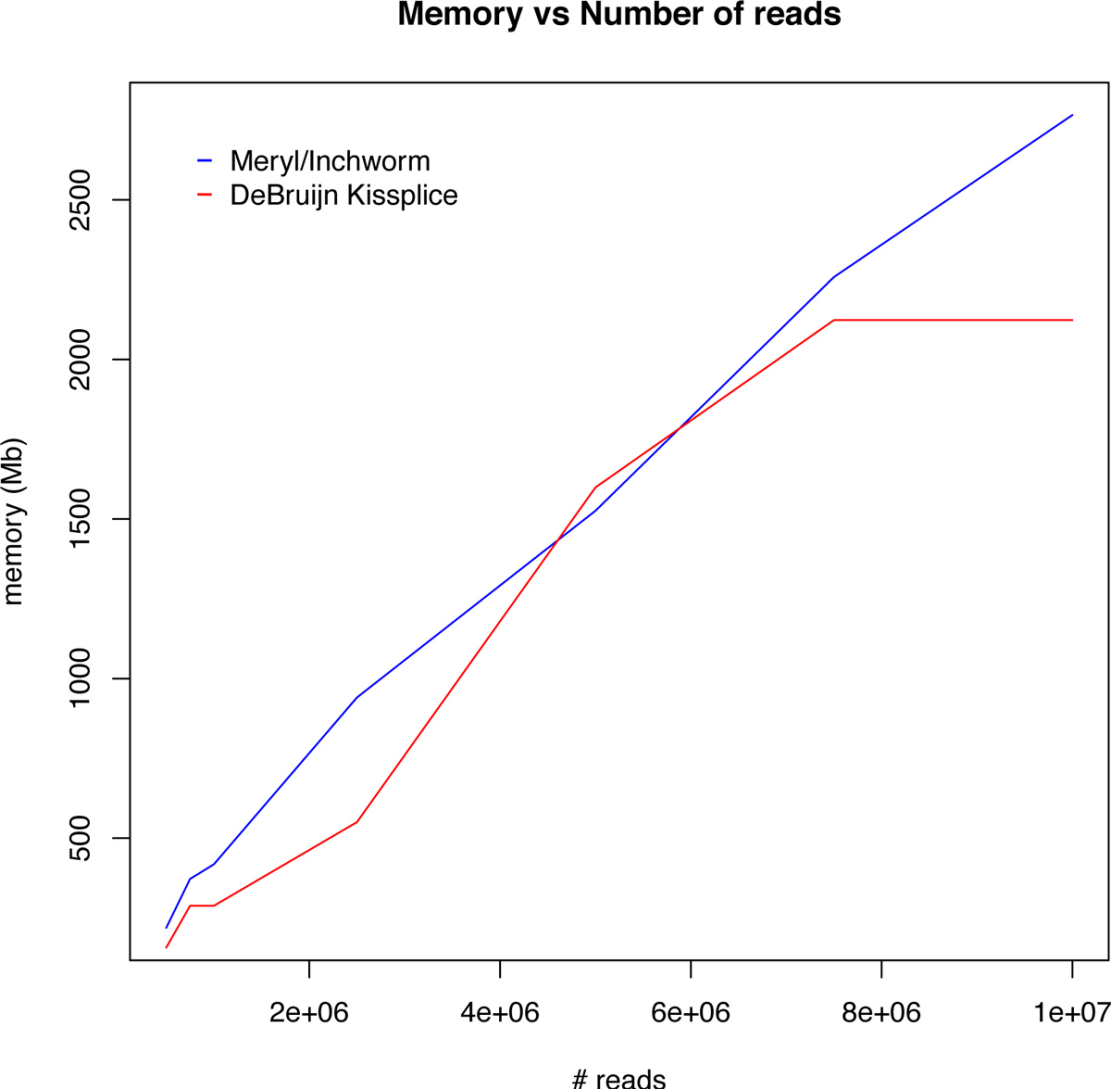
**Memory usage**. Memory usage of KISSPLICE and Inchworm as a function of input size.

**Figure 4:**
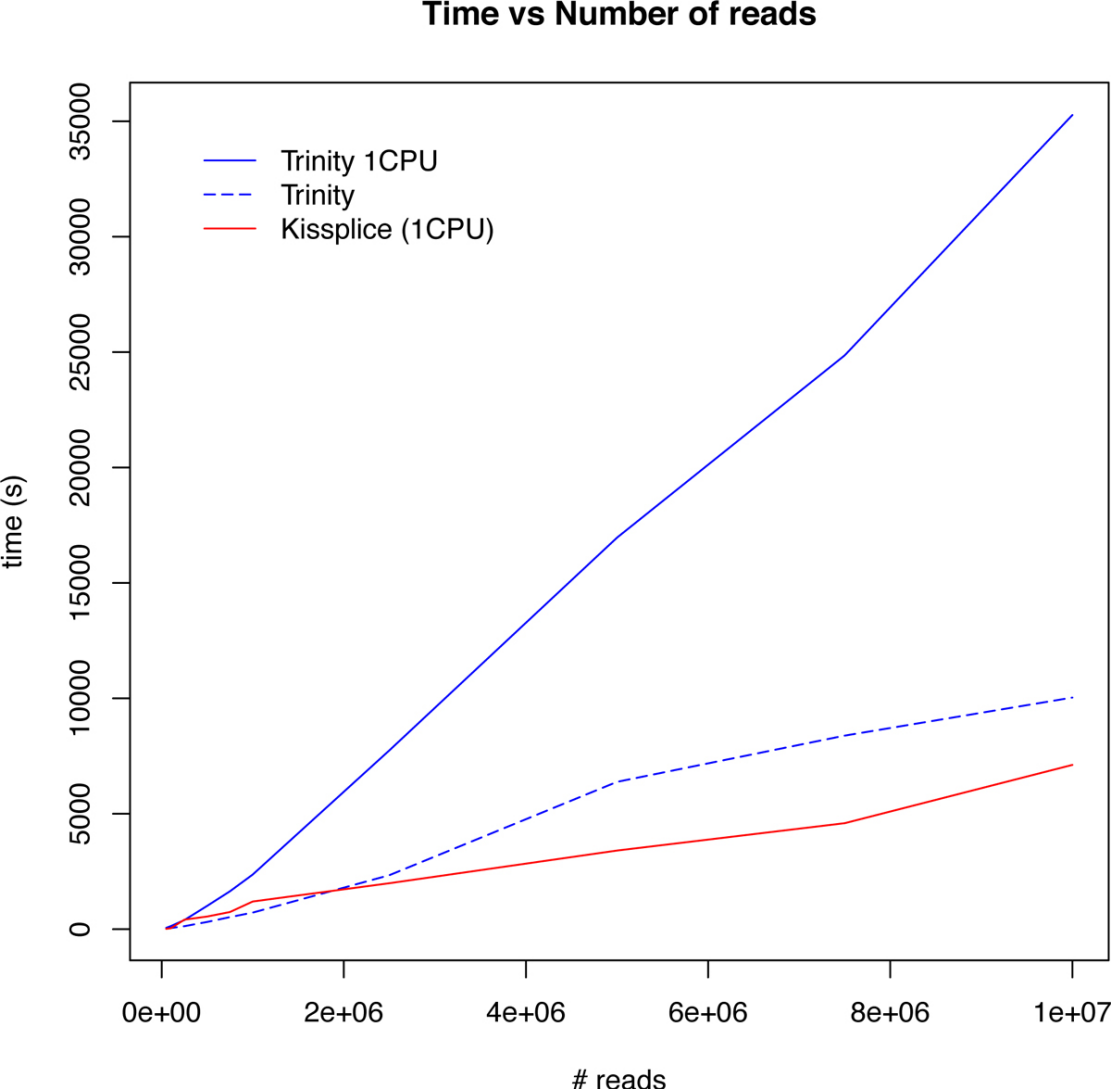
**Time performances**. Time performances of KISSPLICE and TRINITY as a function of input size.

We ran KISSPLICE with *k *= 25 and mkC = 2 on a dataset which consists of 32 M reads from human brain and 39 M reads from liver from the Illumina Body Map 2.0 Project (downloaded from the Sequence Read Archive, accession number ERP000546). As in all DBG-based assemblers, the most memory consuming step was the DBG construction which we performed on a cluster. The memory requirement is directly dependent on the number of unique *k*-mers in the dataset. Despite the fact that we do not use any heuristic to discard *k*-mers from our index, our memory performances are very similar to the ones of Inchworm, the first step of Trinity, as indicated in Figure 3. In addition, for the specific task of calling AS events, KISSPLICE is faster than TRINITY as shown in Figure 4.

KISSPLICE identified 5923 biconnected components which contained at least one bubble, 664 of which consisted of bubbles generated by approximate tandem repeats and 1160 which consisted of bubbles generated by short indels (less than 3 nt). Noticeably, the BCCs which generated most cycles and were most time consuming were associated to approximate tandem repeats. As these bubbles are not of interest for KISSPLICE, this observation prompted us to introduce an additional parameter in KISSPLICE to stop the computation in a BCC if the number of cycles being enumerated reaches a threshold. This enabled us to have a significant gain of time. We however advise not to use this threshold if the purpose is to identify AS events associated to approximate tandem repeats, which we did not address here.

Out of the 4099 remaining BCCs, we found that 3657 were read-coherent (*i*.*e*. each nucleotide is covered by at least one read) and we next focused on this set. For each of the 3657 cases, we tried to align the two paths of each bubble to the reference genome using Blat [18]. If the two paths align with the same initial and final coordinates, then we consider that the bubble is a real AS event. If they align with different initial and final coordinates, then we consider that it is a false positive. Out of the 3657 BCCs, 3497 (95%) corresponded to real AS events, while the remaining corresponded to false positives. A first inspection of these false positives led to the conclusion that the majority of them correspond to chimeric transcripts. Indeed, the shorter path and the longer path both map in two blocks within the same gene, but the second block is either upstream of the first block, or on the reverse strand, in both cases contradicting the annotations and therefore suggesting that the transcripts are chimeric and could have been generated by a genomic rearrangement or a trans-splicing mechanism.

For each of the 3497 real cases, we further tried to establish if they corresponded to annotated splicing events. We therefore first computed all annotated AS events using AStalavista [19] and the UCSC Known Genes annotation [20]. Then, for each aligned bubble, we checked if the coordinates of the aligned blocks matched the splice sites of the annotated AS events. If the answer was positive, then we considered that the AS event we found was known, otherwise we considered it was novel. Out of a total of 3497 cases, we find that only 1538 are known while 1959 are novel. This clearly shows that current annotations largely underestimate the number of alternative transcripts per multi-exon genes as was also reported recently [1].

**Figure 5:**
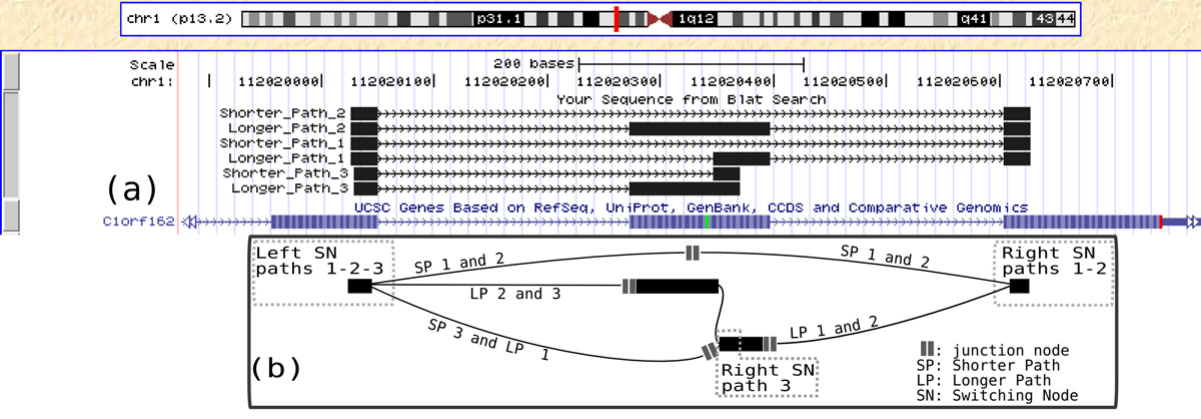
**Complex AS event**. BCC corresponding to a novel complex AS event. The intermediate annotated exon is either present, partially present, or skipped. (a) The annotations (blue track) report only the version where it is present while black tracks report all events found by KISSPLICE. (b) The cDBG associated to this complex event where the junction nodes are composed by 2*k *- 2 nucleotides.

Additionally, we noticed that 719 BCCs contained more than one AS event, which all mapped to the same gene. This corresponds to complex splicing events which involve more than 2 transcripts. Such events have been described in Sammeth et al. [7]. Their existence suggests that more complex models could be established to characterise them as one single event, and not as a collection of simple pairwise events. An example of novel complex AS event is given in Figure 5.

We also found the case where the same AS event maps to multiple locations on the reference genome (423 cases). We think these correspond to families of paralogous genes, which are "collectively" alternatively spliced. We were able to verify this hypothesis on all tested instances. In this case, we are unable to decide which of the genes of the family are producing the alternative transcripts, but we do detect an AS event.

### Characterisation of novel AS events

**Figure 6:**
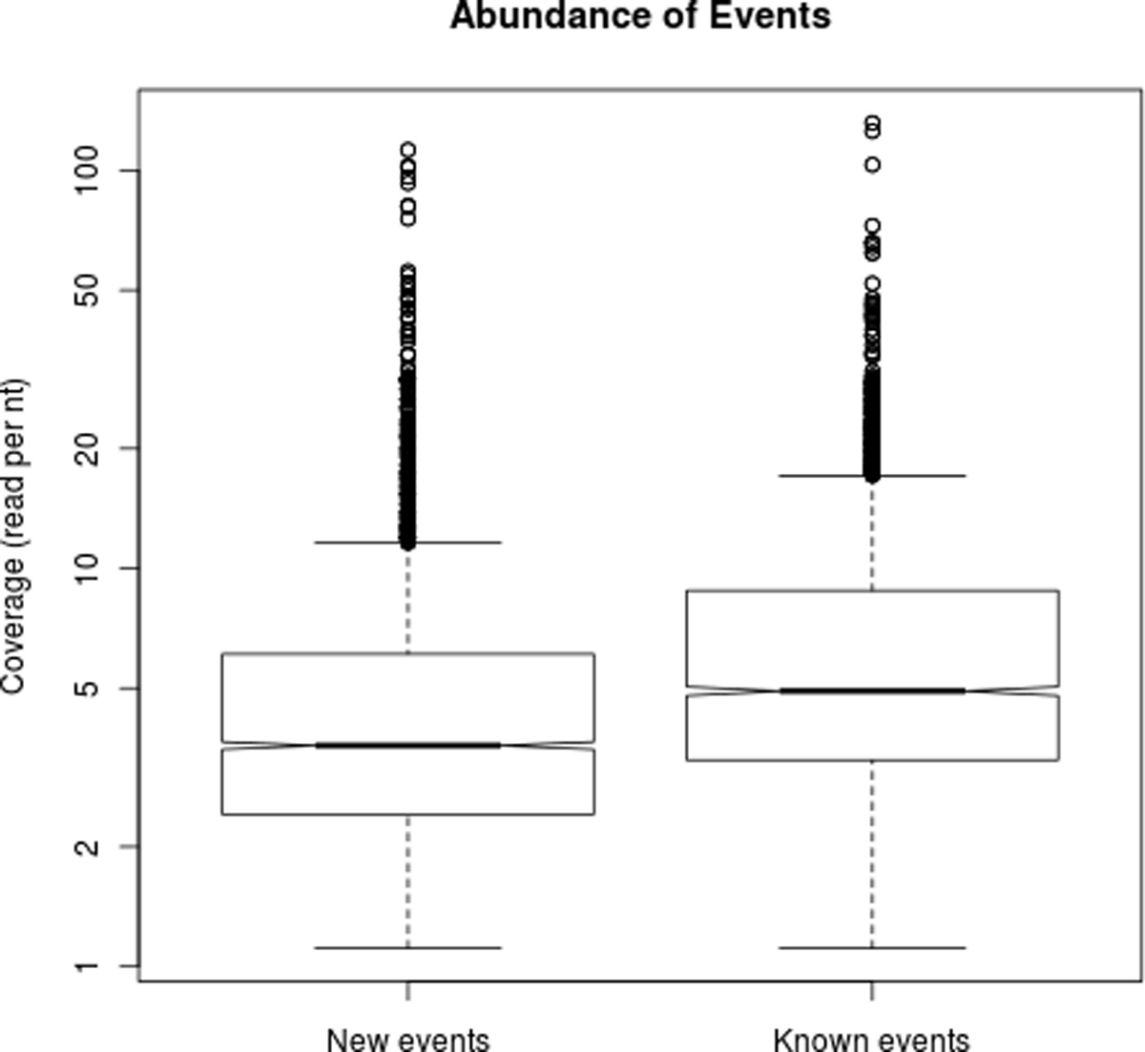
**Event abundances**. Abundance of known and novel events.

In order to further characterise the 1959 novel AS events we found, we compared them with annotated events considering their abundance, length of the variable region and use of splice sites. For each AS event, we have 4 abundances, one for each spliceform (i.e. path of the bubble), and one for each condition. We computed the abundance of an event as the abundance of the minor spliceform. As outlined in Figure 6, we show that novel events are less abundant than annotated events. This in itself could be one of the reasons why they had not been annotated so far. Interestingly, we also found that while annotated events are clearly more expressed in brain than liver (median coverage of 3.4 Vs 1.2), this trend was weaker for novel events (2.4 Vs 1.2). This may reflect the fact that, since tissue-specific splicing in brain has been intensely studied, annotations may be biased in their favour.

**Figure 7:**
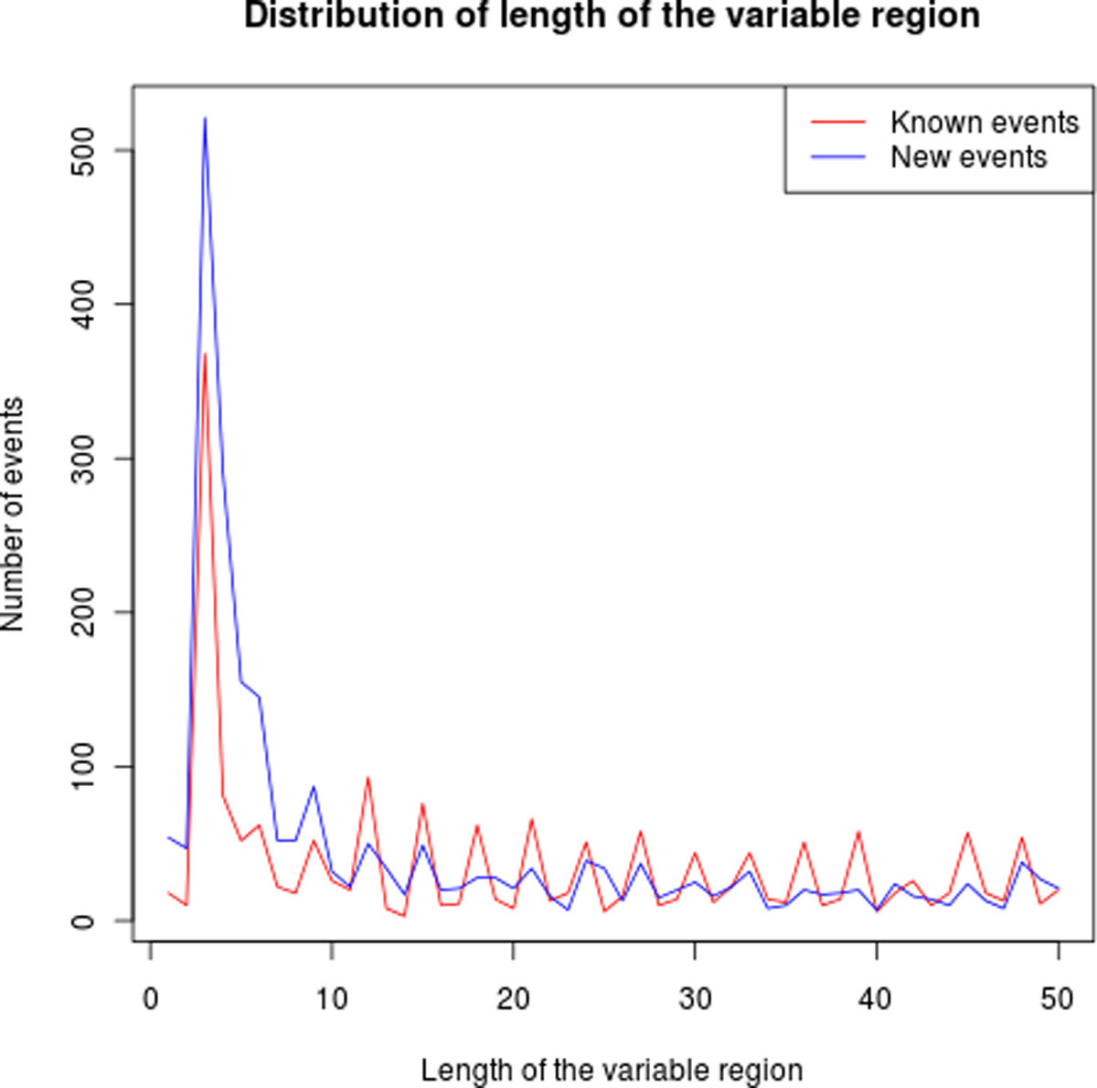
**Event lengths**. Distribution of lengths of the variable regions for known and novel events. Only the initial part of the distribution is given.

We then computed the length of each event as the difference of the length between the two paths of the bubble. We found that for annotated events, there is a clear preference (59%) for lengths that are a multiple of 3, which is expected if the event affects a coding region. However, although still very different from random, this preference is less strong for novel events (45%), which, in addition are particularly enriched in short lengths as shown in Figure 7.

Finally, we computed the splice sites of annotated and novel events, and we found that a vast majority (99.5%) of known events exhibit canonical splice sites, while this is again less strong for novel events (75.3%). Out of the non canonical cases, 13 correspond to U12 introns, but most correspond to short events.

Altogether, while we cannot discard that short non canonical events do occur and have been under-annotated so far, we think that the observations we make on the length and splice site features can be explained by the presence of genomic indels in our results. We had indeed already stated in Section "*De-Bruijn graph models*" that while most annotated genomic indels are below 3 nt, some may still be above. In practice, if the purpose is to strictly study AS events and not indels, then we recommend to focus on events longer than 10 nt, which have canonical splice sites in 95.2% of the cases. More generally, we wish to stress that this confusion between genomic indels and AS events is currently being made by all transcriptome assemblers.

### Comparison with Trinity

Finally, in order to further discuss the sensitivity of our method on real data, we compared our results with TRINITY. Although TRINITY is not tailored to find AS events, we managed to retrieve this information from the output. Whenever TRINITY found several alternative transcripts for one gene, we selected this gene. We further focused on cases which contained a cycle in the splicing graph reconstructed from this gene and we compared them with the events found by KISSPLICE. Whenever we found that both the longer and the shorter path of a bubble were mapping to the transcripts of a TRINITY gene, we decided that both methods had found the same event. In total, KISSPLICE found 4099 cases, TRINITY found 1123 out of which 553 were common. While the sensitivity is overall larger for KISSPLICE, we see that 570 cases are found by Trinity and not by KISSPLICE. We then mapped these transcripts to the human genome using blat. In many instances (348 cases), the transcripts did not align on their entire length, or to different chromosomes, indicating that they corresponded to chimeras. A first inspection of the remaining 222 cases revealed that they correspond to the complex BCCs we chose to neglect at an early stage of the computation, because they contain a very large number of approximate tandem repeats. A first simple way to deal with this issue is to increase the value of *k*. The effect of this is to break the large BCCs into computable cases, enabling to recover a good proportion of the missed events. For instance, for *k *= 35, we found back 84 cases. More generally, this shows that more work on the model and on the algorithms is still required to characterise better AS events which are intricated with approximate tandem repeats. We think that TRINITY manages to identify some of them because it uses heuristics, which enables it to simplify these complex graph structures.

## Conclusions

This paper presents two main contributions. First, we introduced a general model for detecting polymorphisms in De Bruijn graphs, and second, we developed an algorithm, KISSPLICE, to detect AS events in such graphs. This approach enables to tackle the problem of finding AS events without assembling the full-length transcripts, which may be time consuming and uses heuristics that may lead to a loss of information. To our knowledge, this approach is new and should constitute a useful complement to general purpose transcriptome assemblers.

Results on human data show that this approach enables de-novo calling of AS events with a higher sensitivity than obtained by the approaches based on a full assembly of the reads, while using similar memory requirements and less time. 5% of the extracted events correspond to false positives, while the 95% remaining can be separated into known (44%) and novel events (56%). Novel events exhibit similar sequence features as known events as long as we focus on events longer than 10 nt. Below this, novel events seem to be enriched in genomic indels.

KISSPLICE is available for download at http://alcovna.genouest.org/kissplice/ and can already be used to establish a more complete catalog of AS events in many species, whether they have a reference genome or not. Despite the fact that more and more genomes are now being sequenced, the new genome assemblies obtained usually do not reach the level of quality of the ones we have for model organisms. Hence, we think that methods which do not rely on a reference genome are not going to be easily replaced in the near future. There is of course room for future work. The KISSPLICE algorithm could be improved in several ways. The coverage could be used for distinguishing SNPs from sequencing errors. Moreover, the sequences surrounding the bubbles could be locally assembled using a third party tool [21]. This would allow to output their context or the full contig they belong to.

Last, the complex structure of BCCs associated to approximate tandem repeats seems to indicate that more work on the model and on the algorithms is required to efficiently deal with the identification of real approximate tandem repeat events, which may be highly intertwined with other events.

## Competing interests

The authors declare that they have no competing interests.

## Authors' contributions

VL proposed the models, PP GS and MFS developed the algorithms, RC implemented the DBG construction, PA and GS implemented the main algorithms, PP supervised the implementations, JK ran the tests on simulated data, RU ran the tests on real data, VL supervised the tests. All authors contributed to the writing of the paper.
